# The Effect of Hypoxia and Metformin on Fatty Acid Uptake, Storage, and Oxidation in L6 Differentiated Myotubes

**DOI:** 10.3389/fendo.2018.00616

**Published:** 2018-10-17

**Authors:** Martina Musutova, Moustafa Elkalaf, Natalie Klubickova, Michal Koc, Stanislav Povysil, Jan Rambousek, Beatriz Volckaert, Frantisek Duska, Minh Duc Trinh, Martin Kalous, Jan Trnka, Kamila Balusikova, Jan Kovar, Jan Polak

**Affiliations:** ^1^Department for the Study of Obesity and Diabetes, Third Faculty of Medicine, Charles University, Prague, Czechia; ^2^Department of Biochemistry, Cell and Molecular Biology, Third Faculty of Medicine, Charles University, Prague, Czechia; ^3^Department of Anesthesiology and Intensive Care, Third Faculty of Medicine, Charles University, Prague, Czechia; ^4^Department of Cell Biology, Faculty of Science, Charles University, Prague, Czechia; ^5^Division of Cell and Molecular Biology, Third Faculty of Medicine, Department of Biochemistry, Cell and Molecular Biology & Center for Research of Diabetes, Metabolism and Nutrition, Charles University, Prague, Czechia

**Keywords:** hypoxia, myotubes, free fatty acids, FFA oxidation, FFA uptake, metformin, GW501516, CD36 receptor

## Abstract

Metabolic impairments associated with obstructive sleep apnea syndrome (OSA) are linked to tissue hypoxia, however, the explanatory molecular and endocrine mechanisms remain unknown. Using gas-permeable cultureware, we studied the chronic effects of mild and severe hypoxia on free fatty acid (FFA) uptake, storage, and oxidation in L6 myotubes under 20, 4, or 1% O_2_. Additionally, the impact of metformin and the peroxisome proliferator-activated receptor (PPAR) β/δ agonist, called GW501516, were investigated. Exposure to mild and severe hypoxia reduced FFA uptake by 37 and 32%, respectively, while metformin treatment increased FFA uptake by 39% under mild hypoxia. GW501516 reduced FFA uptake under all conditions. Protein expressions of CD36 (cluster of differentiation 36) and SCL27A4 (solute carrier family 27 fatty acid transporter, member 4) were reduced by 17 and 23% under severe hypoxia. Gene expression of UCP2 (uncoupling protein 2) was reduced by severe hypoxia by 81%. Metformin increased CD36 protein levels by 28% under control conditions and SCL27A4 levels by 56% under mild hypoxia. Intracellular lipids were reduced by mild hypoxia by 18%, while in controls only, metformin administration further reduced intracellular lipids (20% O_2_) by 36%. Finally, palmitate oxidation was reduced by severe hypoxia, while metformin treatment reduced non-mitochondrial O_2_ consumption, palmitate oxidation, and proton leak at all O_2_ levels. Hypoxia directly reduced FFA uptake and intracellular lipids uptake in myotubes, at least partially, due to the reduction in CD36 transporters. Metformin, but not GW501516, can increase FFA uptake and SCL27A4 expression under mild hypoxia. Described effects might contribute to elevated plasma FFA levels and metabolic derangements in OSA.

## Introduction

Obstructive sleep apnea syndrome (OSA) is a chronic disorder characterized by periodic upper airway narrowing or complete occlusion during sleep followed by blood hemoglobin desaturation (tissue hypoxemia) and sleep fragmentation. With a prevalence of 5–15% in the general population (1), OSA represents a significant health burden. Several independent studies have identified OSA as a risk factor for hypertension, cardiovascular disease, as well as all-cause mortality, which is independent of other risk factors (2, 3). More recently, studies have indicated that OSA represents a strong risk factor for glucose intolerance, insulin resistance, and type 2 diabetes mellitus (T2DM) when adjusted for confounding variables (e.g., obesity, age, and sex) (4, 5).

Even though cross-sectional as well as prospective studies documented association between OSA and impaired glucose metabolism, the mechanisms mediating this link remain only partially understood (4). Mimicking hemoglobin desaturation observed in OSA patients by exposing humans or rodents to intermittent hypoxia (IH) demonstrated that IH is sufficient to impair fasting and post-challenge glucose levels, diminish insulin sensitivity in muscle tissue and the liver, and reduce pancreatic insulin production (6–10). Among the explanatory molecular mechanisms suggested, activation of the sympathetic nervous system, oxidative stress, stimulation of pro-inflammatory pathways together with increased corticosteroid levels or endothelin-1 signaling have been suggested (4, 11). More recently, interest has focused on the role of circulating free fatty acids (FFA) as a possible mediator of OSA-associated impairments in glucose homeostasis as researchers reported increased FFA levels after intermittent hypoxic exposure in humans and mice (12–14). Importantly, prolonged exposure to elevated plasma FFA levels was shown to induce insulin resistance in muscle and liver tissue (15) as well as impair insulin secretion (16), thus causally contributing to the development of T2DM (17).

Plasma FFA levels are determined by the balance between FFA release from adipose tissue (predominantly through lipolysis) and FFA uptake/oxidation by liver and muscle tissue (18). Although the cause of FFA elevation in the context of OSA and intermittent hypoxia remains to be determined, recent studies have reported that hypoxia stimulates lipolysis in both adipocytes *in vitro* as well as in mouse epididymal adipose tissue (13, 19). Direct measurements of tissue oxygen levels performed during IH (a model of OSA) suggest that skeletal muscle experiences profound hypoxia reaching O_2_ levels of ≈ 26 mmHg (20). The specific effects of hypoxic exposure on FFA uptake and oxidation still remain unclear even though a detailed understanding of FFA turnover in OSA is of significant clinical importance, since lipolysis, as well as FFA oxidation, represent proven pharmacological targets (21). It could be hypothesized, that decreased FFA uptake and/or oxidation in skeletal muscle during hypoxia might contribute to elevated circulating FFA or directly alter intracellular insulin signaling in myocytes.

The aim of this study was to assess the direct effects of mild (4% O_2_) and severe (1% O_2_) hypoxia on FFA uptake, storage, and oxidation in differentiated L6 myotubes. We also investigated, whether pharmacological treatment with metformin or PPAR β/δ agonist could alleviate hypoxia-induced changes in FFA metabolism as metformin was showed previously to reduce intramyocellular lipid accumulation as well to reduce expression of fatty acid transporters and fatty acid oxidation genes in skeletal muscle and liver (22, 23). Similarly, PPAR β/δ activation promotes fatty acid oxidation in skeletal muscle (24). To address these questions, a novel approach utilizing gas-permeable cultureware with a membrane-bottom was employed. This system enables rapid exchange of gases through the membrane, which allows for prolonged exposure of cultured cells to predictable levels of pericellular O_2_ (25, 26).

## Materials and methods

### Cell culture, exposure to hypoxia, and treatment with chemicals

Rat L6-C11 skeletal muscle cells (European Collection of Cell Cultures, Cat. No. 92102119) were expanded up to passage number 10, and subsequently plated in a 24-well fluorocarbon-bottom dishes (Cat. No. 94.6000.014, Sarstedt AG & Co, Nümbrecht, Germany) at a density of 4,000 cells/cm^2^ and cultured in a CO_2_ incubator at 37°C in Dulbecco's Modified Eagle's Medium (DMEM, Cat. No. D6429) supplemented with 10% v/v Fetal bovine serum (FBS, Cat. No. F6178), 1% v/v Penicillin-Streptomycin (Cat. No. P4333), and 1% v/v HEPES (Cat. No. H0887), which was replaced every 48 h until cells reached confluence (7 days). After reaching confluence, concentration of FBS was reduced to 2% to accelerate spontaneous differentiation into myotubes (successful differentiation was evaluated by visual inspection confirming a change in phenotype from individual spindle-like cells to multinucleated tubular structures). Cells were incubated with or without pharmacological treatments and dishes were placed in modular hypoxic incubators (Billups-Rothenberg Inc., Del Mar, CA, USA). Mild and severe hypoxic exposures were achieved by flushing the respective modular incubators with calibration-quality gas mixtures of 4% O_2_ + 5% CO_2_ or 1% O_2_ + 5% CO_2_ (Linde Gas a.s., Prague, Czech Republic). Control exposures were performed in a standard CO_2_ incubator (20% O_2_ + 5% CO_2_). Cells were subsequently cultured for an additional 7 days, with the media being changed every 48 h; the cells were then used for lipid uptake and oxidation assays described below.

The effect of the following drugs during control and hypoxic exposures was investigated: 2 mM metformin (Cat. No. PHR1064), and 100, 500 nM, and 1 μM GW501516 (Cat. No. SML1491). Cells and chemicals were purchased from Sigma-Aldrich (St. Louis, MO, USA).

Control experiments were performed with appropriate vehicles: water for metformin and DMSO (dimethylsulfoxide) for GW501516. Concentrations of GW501516 studied in this paper are reflecting the range of plasma concentrations observed in humans after GW501516 administration (27), although higher (5, 1 μM), as well as lower (100, 10 nM) concentrations were also reported for muscle cells *in vitro* in the published literature (28–31). Similarly, metformin concentration used in this paper are reflecting concentrations typically used in published studies to maximize cellular responses (32–34), while not deviating too far from plasma levels observed in humans treated with metformin, reaching 700 μM (35).

### Determination of fatty acid uptake

Lipid uptake was measured using fluorescently-labeled palmitate (BODIPY® FL C16, Cat. No. D3821 Thermo Fischer Scientific, Waltham, MA, USA). Differentiated cells were starved for 4 h in FBS-free medium. Subsequently, cells were washed and incubated for 3 h under normoxic or hypoxic conditions with or without the previously mentioned pharmacological compounds, in PBS (Dulbecco's Phosphate Buffered Saline, Cat. No. D8662, Sigma-Aldrich, St. Louis, MO, USA) with 1 μM of fluorescently-labeled palmitate (BODIPY FL® C16, stock prepared in DMSO) and 0.1% fatty acid free BSA (bovine serum albumin, Cat. No. A7030 Sigma-Aldrich, St. Louis, MO, USA). After incubation, cells were washed twice with 1 mL PBS and lysed in 150 μL of T-PER (Tissue-Protein Extraction Reagent, Cat. No. RL243205 Thermo Fischer Scientific, Waltham, MA, USA). Fluorescence of intracellular BODIPY-labeled palmitate was measured with an Infinite® 200 PRO (Tecan Trading AG, Switzerland) microplate reader with excitation/emission wavelengths of 470/503 nm. Data were normalized to protein concentration in each well, which was measured using a BCA assay (bicinchoninic acid assay, Cat. No. 23225 Thermo Fischer Scientific, Waltham, MA, USA).

### Determination of intracellular lipid stores

Intracellular lipid stores were quantified using the fluorescence lipid staining method. Cells were fixed for 1 h in 10% formalin, washed twice with 1 mL PBS, and stained with a working solution of 1 μg/mL BODIPY 493/503 (Cat. No D3922, Thermo Fischer Scientific, Waltham, MA, USA) for 30 min. Subsequently, cells were washed, lysed with T-PER and fluorescence entrapped by the cells was determined using an Infinite®200 PRO microplate reader with excitation/emission wavelengths 493/503 nm. Data are expressed as relative values compared to control conditions.

### Determination of fatty acid oxidation

Cells were homogenized in 10% sucrose using a motor-driven homogenizer at 800 rpm. Oxygen uptake in homogenized samples was measured using a High-Resolution Oxygraph-2K instrument (Oroboros, Innsbruck, Austria). Measurements were performed at 30°C in 2 mL of incubation medium containing 1 mM EDTA, 75 mM KCl, 5 mM KH_2_PO_4_, 3 mM MgCl_2_ 6 H_2_O, and 8 mM Tris HCl, at pH 7.4. The sequence of substrate/drug additions to the measuring chamber was as follows: 1 mM malate, 1.5 mM ADP (adenosine triphosphate), 5 μM carnitine palmitoyl followed by 0.1 mM etomoxir (inhibiting FFA mitochondrial transport), 1 μM oligomycin (inhibiting ATP-synthase), and last, 4 μM antimycin A (an electron transport chain inhibitor). At each step, oxygen uptake was measured until it reached a plateau. The rate of oxygen uptake was expressed as pmol/s/mg protein determined using a BCA assay (Cat. No. 23225 Thermo Fischer Scientific, Waltham, MA, USA).

### Co-localization analysis

Cells were fixed in 4% formaldehyde for 15 min and washed with PBS. Subsequently, the nuclei were stained with Hoechst (1:300 in PBS for 15 min), lipids were stained for 2 h in 1 μg/mL BODIPY 493/503 (Prod. No. D3922, Thermo Fisher Scientific, Waltham, MA, USA) and cells were washed 3 times with PBS. Lastly, cells were permeabilized for 5 min using 0.2% Triton X (Prod. No. X100, Sigma Aldrich, St. Louis, MO, USA) and (1) incubated for 15 min with 5 μg/mL of Lectin HPA Alexa Fluor® 647 conjugate (Prod. No. L32454, Thermo Fisher Scientific, Waltham, MA, USA) to visualize the Golgi apparatus or (2) blocked with 1% BSA (bovine serum albumin, Prod. No. A7030, Sigma Aldrich, St. Louis, MO, US) for 1 h and incubated overnight with 1:100 anti-PMP70 (The 70-kDa peroxisomal membrane protein) rabbit monoclonal antibody conjugated with Alexa Fluor® 647 (ab199019, Abcam, Cambridge, UK) to visualize peroxisomes. Nuclei counterstaining was performed using Hoechst 33258 (Prod. No. H1398, Thermo Fisher Scientific, Waltham, MA, USA). Images were captured using a Leica TCS SP5 confocal microscope (Leica Microsystems, Wetzlar, Germany) and digitally processed using Leica Software. Co-localization analysis was performed and expressed as an overlap coefficient.

### Gene and protein expression analysis

Isolated RNA was treated with DNAse (Roche Diagnostics, Mannheim, Germany) and gene expression of cluster of differentiation 36 (CD36), solute carrier family 27 fatty acid transporter, member 4 (SCL27A4), carnitine palmitoyltransferase I (CPT1), peroxisome proliferator-activated receptor gamma coactivator 1-alpha (PGC1α), uncoupling protein 2 (UCP2) glucuronidase beta (GUSB), and TATA box binding protein (TBP) was assessed using quantitative PCR (qPCR) (Applied Biosystems, Carlsbad, CA) using TaqMan probes (Product ID: Rn00580728_m1, Rn01438951_m1, Rn00682395_m1, Rn00580241_m1, Rn01754856_m1, Rn00566655_m1, Rn01455646_m1). Differences in relative gene expression was assessed using the REST software (Qiagen, Hilden, Germany) incorporating the Pfaffl's bootstrapping algorithm.

Western blotting: Protein expression of SLC27A4, CD36, and GAPDH (Glyceraldehyde 3-phosphate dehydrogenase—a loading control) was performed by western blot analysis using rabbit polyclonal antibodies (Anti-SLC27A4 antibody: ab199718, Anti-CD36 antibody: ab133625, and Anti-GAPDH antibody: ab9485, all from Abcam, Cambridge, UK). Goat anti-rabbit IgG antibody conjugated with a horseradish peroxidase (sc-2004, Santa Cruz Biotechnology (Dallas, Texas, USA) was used as a secondary antibody. Denatured proteins were separated electrophoretically on an SDS/12% polyacrylamide gel at 125 V using a Criterion Cell (Bio-Rad, Hercules, CA, USA) and then blotted onto a 0.2 μm nitrocellulose membrane for 1.5 h at 100 V, using a Criterion Wire Blotter System (Bio-Rad, Hercules, CA, USA). The membranes for SLC27A4 and GAPDH quantification were blocked with 5% BSA in TBS (100 mM Tris-HCl, 150 mM NaCl, pH = 7.5) for 30 min, the membrane for CD36 was blocked with 5% non-fat milk in TBS for 30 min. After washing with TBST (TBS + 0.1% Tween-20), the membranes were incubated with the relevant primary antibody and incubated for 2 h with the corresponding secondary antibody. After washing, detection was performed using enhanced chemiluminescence method with SuperSignal West Pico PLUS Chemiluminescent Substrate (Pierce, Rockford, IL, USA) and a Gel Logic 4000 PRO Imaging System (Carestream Health, New Haven, CT, USA). Band intensities were quantified using Image J software (National Institutes of Health, Bethesda, USA). Band intensities of CD36 and SLC27A4 were normalized to GAPDH signal.

### Statistical analysis and calculations

The effect of hypoxia on the outcome variables was analyzed using the ANOVA test with Tukey's *post-hoc* analysis using GraphPad (GraphPad Software, Inc., La Jolla, CA, USA). The effect of the pharmacological substances on the outcome variables under various O_2_ levels was analyzed using the 2-way ANOVA and interactions between pharmacological treatment and O_2_ levels was determined. Data are presented as mean ± SEM. A value of *p* < 0.05 was considered significant.

## Results

### The effect of hypoxia, metformin, and PPAR β/δ agonist on FFA uptake in L6 myotubes

**Figure 1:**
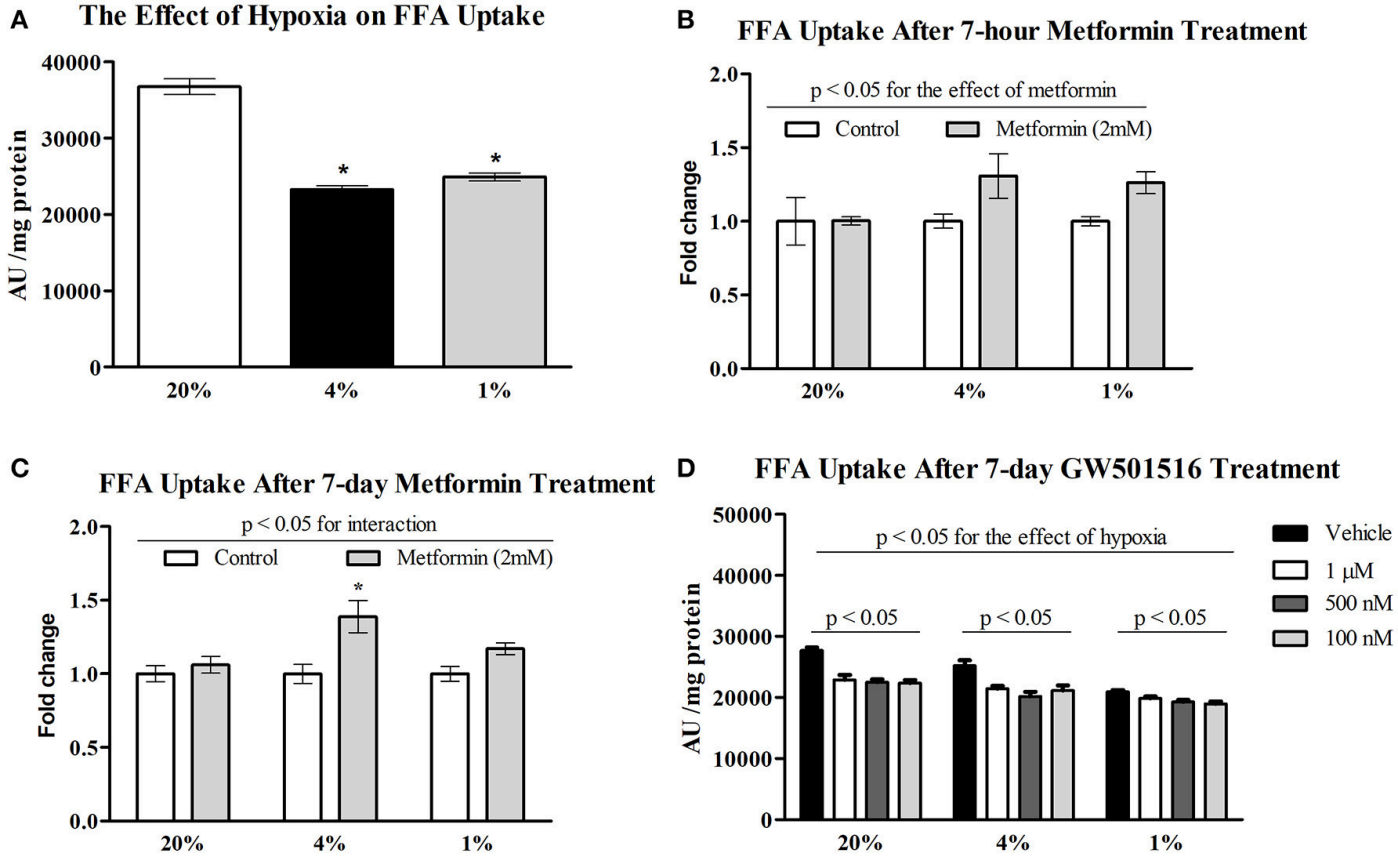
Effect of hypoxia on FFA uptake relative to metformin and GW501516 treatment. The effect of 4% O_2_ and 1% O_2_ hypoxia on FFA uptake **(A)** the impact of acute **(B)** and prolonged **(C)** 2 mM metformin administration on FFA uptake; and the effect of various concentrations of GW501516 (a PPAR β/δ agonist) on FFA uptake **(D)**. ^*^p < 0.05 for comparison with control exposures (20% O_2_, ANOVA), *n* = 12 **(A,C)**, *n* = 6 **(B,D)**. Two-way ANOVA was used to explore interaction between exposure to hypoxia and metformin treatment.

Exposure to pericellular O_2_ levels of 4 and 1% for 7 days decreased FFA uptake in differentiated L6 myotubes by 37% (36718 ± 3585 vs. 23249 ± 1810 AU/mg protein, *p* < 0.001) and 32%, respectively (36718 ± 3585 vs. 24922 ± 1822 AU/mg protein, *p* < 0.001). There was no additive effect associated with severe (1%) compared to moderate (4%) hypoxia on FFA uptake (Figure 1A). Acute treatment with 2 mM metformin had no effect on FFA uptake at 20% O_2_, however 7-h metformin administration increased FFA uptake at 4% O_2_ and 1% O_2_ by 31 and 26% (Figure 1B). Similarly, 7-day metformin treatment increased FFA uptake by 39 and 17% respectively; however, a significant interaction between hypoxia and metformin treatment was only achieved after 7-days of metformin treatment (2-way ANOVA, *p* < 0.05 for interaction), Figure 1C. As a result, 7-day treatment was further investigated in subsequent analyses. Treatment with 100, 500 nM, and 1 μM GW501516 (PPAR β/δ agonist) reduced FFA uptake under control and hypoxic conditions (Figure 1D).

### The effect of hypoxia and metformin on gene and protein expression of FFA transporters

**Figure 2:**
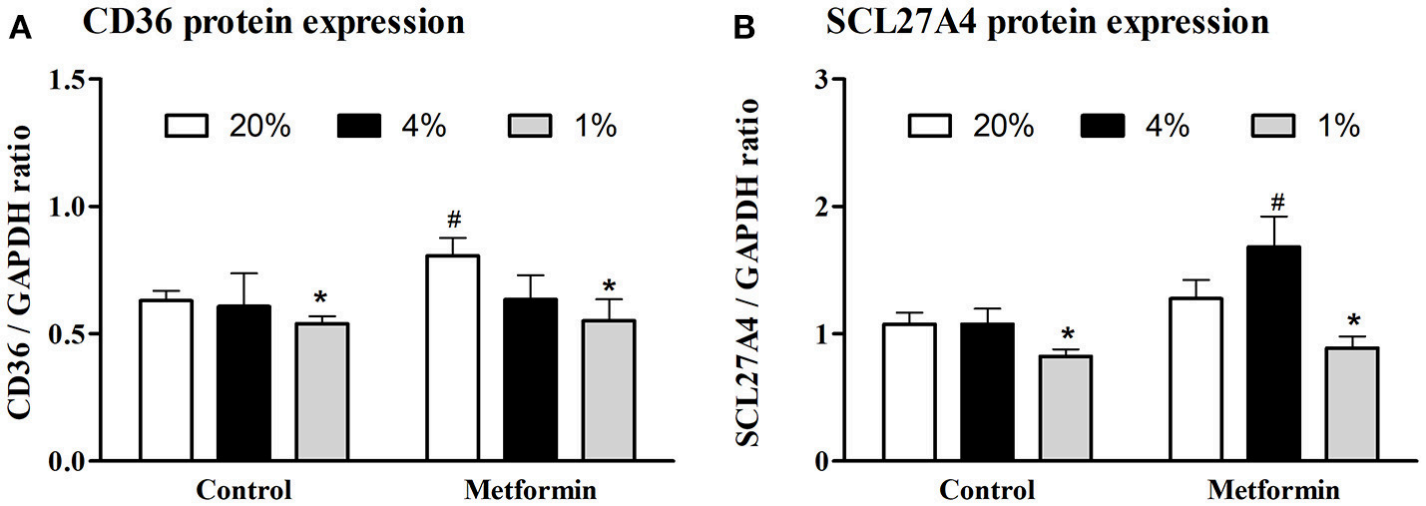
Protein expression of FFA protein transporters. The effect of mild (4% O_2_) and severe (1% O_2_) hypoxia **(A)** and metformin administration **(B)** on CD36 and SCL27A4 protein expression. ^*^p < 0.05 for comparison with control exposures (20% O_2_, unpaired *T*-test), ^#^*p* < 0.05 for comparison with vehicle-treated group (unpaired *T*-test), *n* = 6–9.

**Figure 3:**
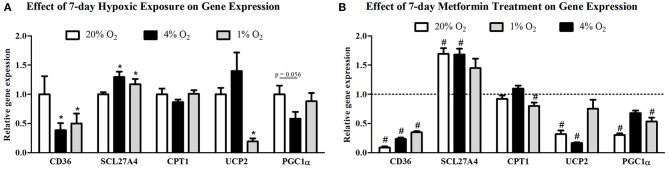
Gene expression of FFA protein transporters and key regulators of mitochondria metabolism. The effect of mild (4% O_2_) and severe (1% O_2_) hypoxia **(A)** and metformin administration **(B)** on CD36, SCL27A4, CPT1, UCP2, and PGC1α gene expression. ^*^p < 0.05 for comparison with control exposures (20% O_2_), ^#^*p* < 0.05 for comparison with vehicle-treated group, *n* = 6. Gene expression data were analyzed using the REST Software (Qiagen, Hilden, Germany) based on the Pfaffl's method.

Protein expression of CD36 transporter was not affected by mild hypoxia (0.63 ± 0.04 vs. 0.61 ± 0.13, *p* > 0.05), but dropped by 17% under severely hypoxic (1% O_2_) conditions (0.63 ± 0.04 vs. 0.52 ± 0.03, *p* < 0.05), Figure 2A. Identical pattern was observed for SCL27A4 protein expression, remaining unchanged after mild hypoxia (1.08 ± 0.09 vs. 1.08 ± 0.12, *p* > 0.05) but decreasing with severe hypoxic exposure (1.08 ± 0.09 vs. 0.82 ± 0.06, *p* < 0.05), Figure 2B. Gene expression of fatty acid transporters was differentially modulated by hypoxia in L6 myotubes, while expression of CD36 decreased by 61% and 50% (both *p* < 0.05) after exposure to 4 and 1% O_2_, expression of SCL27A4 increased by 30 and 17% (both *p* < 0.05) in 4 and 1% O_2_, respectively, Figure 3A. Exposure to 1% O_2_ (but not 4% O_2_) also decreased expression of mitochondrial uncoupling protein UCP2 by 81% (*p* < 0.05), Figure 3A. Data are summarized in Supplementary Material Table 1.

Metformin treatment increased FFA transporter SCL27A4 protein expression by 55% (1.08 ± 0.12 vs. 1.68 ± 0.24, *p* < 0.05) (Figure 2B) as well as its gene expression by 30% (*p* < 0.05) (Figure 3B) under control conditions and mild hypoxia, while gene expression of CD36 was reduced by 91, 75, and 65% (all *p* < 0.05) with metformin treatment under 20, 4, and 1% O_2_, respectively (Figure 3B). Protein expression of CD36 was moderately elevated by 28% with metformin administration under control conditions only (0.63 ± 0.04 vs. 0.81 ± 0.07, *p* < 0.05) (Figure 2A). Metformin administration also reduced gene expression of PGC1α and UCP2 under control conditions and under 4% O_2_ (UCP2) and 1% O_2_ (PGC1α). Gene expression of the key regulator of FFA oxidation (CPT1) remained unaffected by hypoxia or metformin treatment (Figures 3A,B). Data and Western blot pictures are provided in Supplemental Data Sheet 1.

### The effect of hypoxia and metformin on FFA oxidation

**Figure 4:**
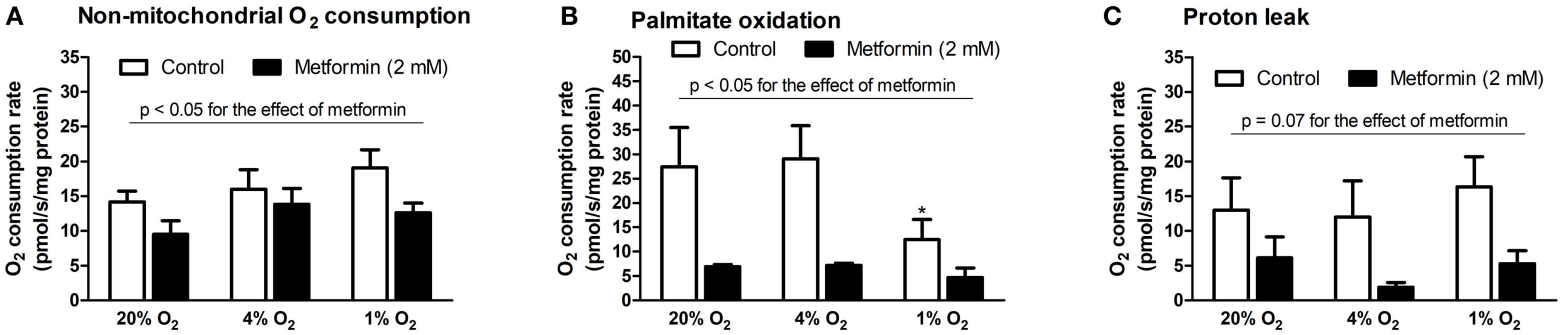
Mitochondrial respiration analysis during hypoxia with metformin administration. **(A)** Non-mitochondrial respiration (residual O_2_ consumption rate after addition of oligomycin), **(B)** palmitate oxidation (O_2_ consumption rate after addition of palmitoyl-carnitine subtracted from O_2_ consumption rate after addition of etomoxir), **(C)** mitochondrial proton leak (O_2_ consumption rate after addition of antimycin A subtracted from O_2_ consumption rate after addition of oligomycin). ^*^p < 0.05 for comparison with control exposures (20% O_2_), *n* = 8 (control conditions), *n* = 4 (metformin administration). Two-way ANOVA was used to explore interaction between exposure to hypoxia and metformin treatment.

The influence of hypoxia on mitochondrial and non-mitochondrial O_2_ consumption was investigated in L6 myotube lysates. As summarized in Figure 4, severe hypoxia (1% O_2_) reduced palmitate oxidation (27.5 ± 8.3 vs. 12.5 ± 4.2 pmol/s/mg protein, *p* = 0.05, Figure 4B), however, other parameters were not affected by pericellular O_2_ levels. In contrast, metformin treatment reduced non-mitochondrial O_2_ consumption, palmitate oxidation as well as proton leak under at all tested O_2_ levels, Figures 4A,C.

### The effect of hypoxia and metformin on intracellular lipid stores

**Figure 5:**
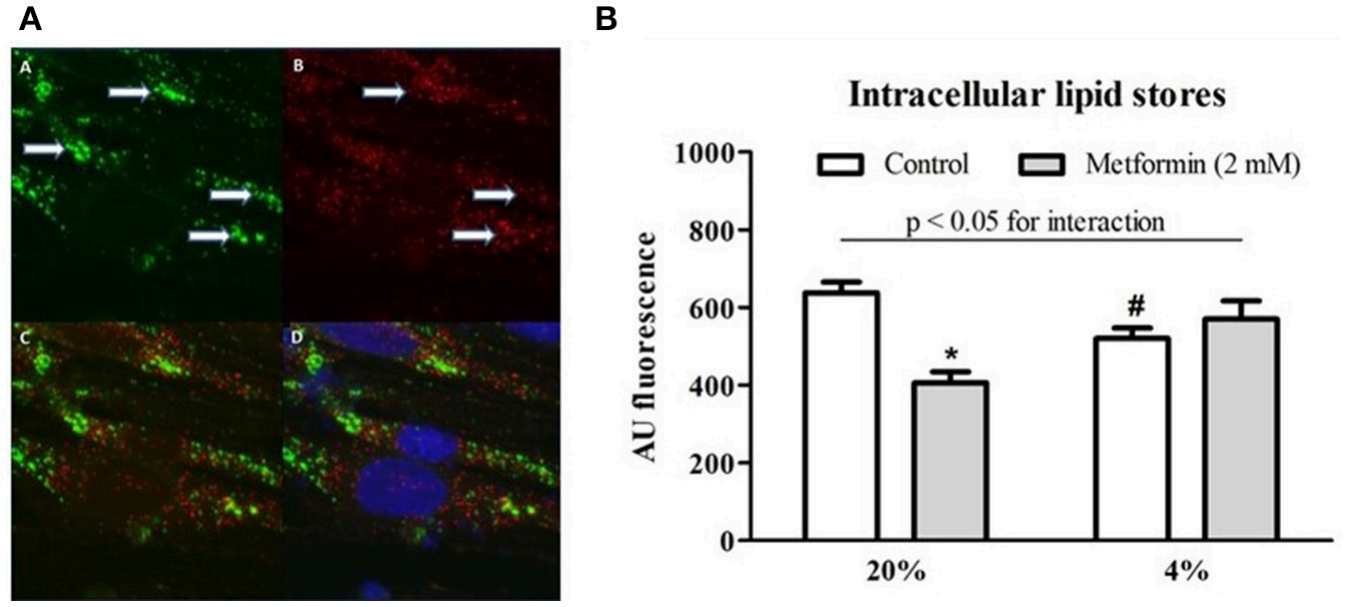
Lipid co-localization imaging and total intracellular lipid content. **(A)** A representative example of a microscope image of lipids stained with BODIPY 493/503 (green) forming distinct vesicular structures marked by arrows (A), staining (red) for peroxisome-specific protein PMP70 (B), merged image of lipid and PMP70 staining (C) and merged image of lipid, PMP70, and nuclei staining (blue) with Hoechst 33258 (D). **(B)** Total lipid content during hypoxia and after metformin treatment The effect of hypoxic exposure (4% O_2_) and 7-day metformin administration on total lipid content in differentiated L6 myotubes. ^*^p < 0.05 for comparison with vehicle-treated cells, ^#^*p* < 0.05 for comparison with control exposures (20% O_2_), *n* = 6. Two-way ANOVA was used to explore interaction between exposure to hypoxia and metformin treatment.

Intracellular lipids were localized in two forms within L6 myotubes, as shown in Figure 5A: (1) as diffusely spread throughout the cytoplasm and (2) concentrated inside cytoplasmic vesicles positively staining for PMP70 (a 70-kDa peroxisomal membrane protein). Exposure to 4% O_2_ reduced total intracellular lipid stores by 18% (AU: 638 ± 27.5 vs. 520.3 ± 26.4, *p* < 0.05). Metformin administration decreased total lipid content under control conditions (20% O_2_) by 36% (AU: 638 ± 27.5 vs. 406.6 ± 28.2, *p* < 0.05); however, no effect of metformin was observed under hypoxic conditions (AU: 520.3 ± 26.4 vs. 570.8 ± 46.4, *p* > 0.05), Figure 5B. Less lipids were localized in peroxisomes under 1% O_2_ and 4% O_2_ hypoxia compared to control conditions (overlap coefficient: 0.66 ± 0.02 and 0.68 ± 0.03 vs. 0.74 ± 0.01, respectively, *p* < 0.05), while co-localization of lipids with the Golgi apparatus remained unchanged (overlap coefficient: 0.58 ± 0.03, 0.46 ± 0.08, and 0.54 ± 0.04 for 20, 4, and 1% O_2_, respectively, *p* > 0.05).

## Discussion

The present study utilized a validated membrane-bottom cultureware technology (25), enabling long-term exposure of adherent cells to desired oxygen levels in the pericellular space, to assess the effects of moderate (4% O_2_) and severe (1% O_2_) hypoxia on lipid uptake and metabolism in differentiated L6 myotubes. We observed that both moderate and severe hypoxia reduced FFA uptake and intracellular lipid stores in peroxisomes, which was probably due the inability to oxidize FFA under hypoxic conditions together with mild reduction in FFA transporter proteins (in severe hypoxia). Metformin administration for 7 days increased FFA uptake, increased gene expression of the FFA transport protein, SCL27A4, and reduced palmitate oxidation, which prevented a hypoxia-induced decline in intracellular lipid stores.

The overarching goal of this study was to assess the role of modified FFA metabolism in skeletal muscle as a possible causative mechanism mediating the established link between obstructive sleep apnea syndrome (OSA) and the development of type 2 diabetes (4, 36, 37). The present study provided two potential mechanisms for hypoxia-induced metabolic derangements: first, decreased FFA uptake in myotubes exposed to hypoxia might, together with increased lipolysis in adipose tissue (19, 13), contribute to elevated levels of FFA in OSA patients (38) and lead to development of pancreatic β-cell dysfunction and insulin resistance in liver and muscle (17, 39, 40). Second, reduced FFA oxidation, which was observed under severe hypoxia, represents a feature typically observed in obese and type 2 diabetes subjects causally associated with increased intracellular lipid content, impaired insulin signaling, and reduced glucose uptake (41–44). *In-vitro* studies of cultured cells have proved useful in OSA research since they allow for investigation of the direct effects of hypoxia separated from the interference of other factors typically observed in OSA, e.g., sleep fragmentation, endocrine adaptations, and autonomic nervous system contributions (4). Direct tissue oxygen levels recordings in mice revealed that under conditions equivalent to severe OSA with 60 hypoxic episodes per hour, skeletal muscles becomes profoundly and nearly continually hypoxic with tissue O_2_ levels ranging from ≈ 2 to 5% O_2_ (20). The levels of mild and severe hypoxia (4 and 1% O_2_), as defined in the present study, not only reflect tissue O_2_ levels expected in severe OSA, but also allow for assessment of mechanisms associated with hypoxia-inducible factor 1 (HIF-1) activation, which starts at O_2_ levels of 4–5% and reaches a maximum at 0.5% O_2_ (45, 46).

Uptake of FFA across the sarcolemma membrane happens partially through passive diffusion, however, its facilitation by protein transporters (e.g., CD36, FABPpm, and SCL27A4) has been demonstrated in various cell types including heart and muscle, together with their regulation by physiological stimuli (47–50). As oxygen availability significantly modulates energy metabolism (51), several groups investigated the effect of hypoxia on expression and localization of FFA transporters. In fact, it has been shown that acute hypoxic exposure increased CD36 expression, plasma membrane localization and FFA uptake in cardiomyocytes (52) and decreased expression of SCL27A4 in placenta (53). Furthermore, it has been shown that CD36 levels are acutely regulated by HIF-1 dependent mechanisms (54). In contrast, human experiments documented that acute hypoxia increased circulating FFA levels (55, 56). Although our observations contradict some of these reports, it needs to be emphasized that our study (employing a gas-permeable cultureware) investigated the response of cells to a prolonged, 7-day, hypoxic exposure (as compared to minutes or hours of hypoxia in the mentioned studies). It is plausible to hypothesize, that acute hypoxia-induced changes differ markedly from adaptive, long-term, effects of hypoxia. In fact, up-regulation of HIF-1 by hypoxia shows a transient pattern with a peak activation detected few hours after the hypoxic exposure followed by a long-term decline (57–59). Additionally, reduced FFA uptake in hypoxic myotubes is congruent with reports demonstrating a significant reduction in skeletal muscle FFA uptake and oxidation in humans under hypoxic conditions (60, 61). These effects have been associated with reduced mitochondrial mass, CPT-1 (carnitine-palmitoyl transferase-1), PPARα and various tricarboxylic acid cycle enzymes activity or expression in hypoxic skeletal muscle (62–65), and reflect the metabolic feature of FFA, which is that they can only be oxidized by aerobic phosphorylation. Based on our observations, hypoxia did not reduce expression of key mitochondrial FFA transporter (CPT1) nor did it significantly reduce expression of mitochondrial biogenesis and metabolism regulator (PGC1α), suggesting that limited O_2_ availability *per se* might reduce FFA oxidation. Shift toward glycolytic energy metabolism in severe hypoxia is further supported by observed reduction of UCP2 gene expression which was previously shown to limit aerobic phosphorylation and increase dependence on glucose as energy substrate (66, 67). Similarly to data previously reported in hypoxic adipocytes (68), we also observed that severe hypoxia reduced protein expression of CD36 which might further contribute to decreased FFA uptake and extended this finding to another important FFA transporter SCL27A4. However, as we did not quantify the membrane localization of both transporters, relevant for the FFA transport (69), it is possible that their contribution was underestimated due to their possible reduced localization in the plasma membrane. Importantly, differential response of both FFA transporters was observed after metformin administration which increased SCL27A4 protein expression under mild hypoxia, while both transporters were reduced by metformin in severe (1% O_2_) hypoxia.

Impaired glucose homeostasis observed in OSA subjects was subsequently reproduced in mice exposed to intermittent hypoxia, suggesting the key role of hypoxia. However, mice studies, as well as a recent randomized clinical trial in diabetic OSA patients, reported only limited reversibility of hypoxia-induced metabolic impairments by cessation of hypoxic exposure or OSA treatment (7, 70). These observations, combined with unsatisfactory compliance with continuous positive airway pressure (CPAP) treatment in OSA patients (71), warrants the search for possible pharmacological treatments. In the present study, the ability of metformin and GW501516 (a selective PPARβ/δ receptor agonist) to alleviate hypoxia-induced derangements in FFA metabolism was assessed. From the tested chemicals, metformin increased, while GW501516 reduced FFA uptake, even though previous reports showed the ability of GW501516 to stimulate FFA oxidation in muscle cells (29). As FFA uptake and oxidation represent two distinct processes, it can be hypothesized that GW501516 might increase FFA oxidation and, at the same time, reduce FFA uptake—this would reduce intracellular lipid stores and possibly improve cellular insulin sensitivity. In the subsequent studies, we considered GW501516 to be a suboptimal pharmacological candidate for the treatment of reduced FFA uptake (as it further reduced FFA uptake) and thus subsequently evaluated the mechanistic consequences behind the metformin effect. The results showed that metformin administration increased FFA uptake under hypoxic conditions, partially through the up-regulation of SCL27A4, however, other mechanisms, e.g., increased FFA uptake mediated by activation of AMP-activated protein kinase (AMPK) (72–74), are probably also involved. Besides the AMPK-mediated effects, metformin was identified as a potent inhibitor of mitochondrial respiratory chain complex I activity, which reduces mitochondrial capacity to oxidize substrates, e.g., malate and glutamate (75, 76). Similarly, we observed reduced palmitate oxidation and less proton leak after chronic (7 days) metformin administration, which is congruent with the limited ability to oxidize acetyl-CoA when complex I is inhibited by metformin. Although a reduction in FFA oxidation might contribute to increased intracellular lipid stores and potentially impair glucose uptake in muscles, the opposite effects have been repeatedly and independently described. For example, reduction of FFA oxidation with etomoxir increased intracellular lipid stores but at the same time enhanced insulin sensitivity, GLUT4 membrane translocation and glucose uptake in various models including humans, mice, and *in-vitro* experiments (76–78). Indeed, hypoxia in humans has been associated with a nearly doubled glucose uptake by muscles despite lowered FFA oxidation (55). Further investigation of the effects of prolonged hypoxia on insulin sensitivity in myotubes (e.g., insulin-stimulated glucose uptake, quantification of pAKT/AKT, or detection of insulin responsiveness genes) is needed to elucidate metabolic status of myocytes under hypoxia. Although these important variables were not measured in our study, previous reports showed that exposure of L6 myotubes to 1% O_2_ increased expression of GLUT1 by ~30% together with a 5-fold induction of insulin-independent glucose uptake. Surprisingly, glucose uptake under hypoxia was not stimulated by insulin administration, even though levels of IRβ (Insulin receptor-β) and IRS-1 (insulin receptor substrate-1) proteins remained unchanged (79). Additionally, protein levels, phosphorylation status and activity of AKT/pAKT were lowered by 24 h exposure to 1% O_2_ (80).

Intracellular localization of lipids was also investigated in the present paper to better understand intracellular lipid handling and the impact of hypoxia. We showed that intramyocellular lipids were distributed diffusely in the cytoplasm but also formed distinct vesicular structures that stained for peroxisome-specific protein PMP70. Although peroxisomes exert multiple functions, FFA β-oxidation ranks among the most preserved functions of peroxisomes across species (81). Hypoxia reduced intracellular lipid stores as well as reduced lipid localization into peroxisomes suggesting a reduction in peroxisome lipid content. These observations are in line with previous reports of inhibited peroxisome biogenesis and increased degradation of peroxisomes as a result of hypoxic exposure and hypoxia inducible factor 2α (HIF-2α) activation (82, 83). We speculate that reduction in peroxisome lipids might explain the observation of reduced total intracellular lipid content, however, further investigation involving quantitative analysis of total peroxisome abundance is warranted.

In summary, this study showed that exposure to hypoxia reduced FFA uptake in L6 differentiated myotubes, partially due to a reduction in the CD36 and SCL27A4 FFA transporters, which might contribute to elevated circulating FFA and subsequently to the development of T2DM associated with OSA. We also demonstrated the potential of metformin to alleviate hypoxia-induced impairments through increasing FFA uptake and by reducing FFA oxidation in mitochondria, thus enabling higher glucose uptake and oxidation. The present study provides a mechanistic background that partially elucidated the links between OSA and associated metabolic impairments including type 2 diabetes mellitus.

## Author contributions

MM experiments investigating FFA uptake in normoxic and hypoxic conditions, Oxygraph measurements of palmitate oxidation, sample collection for gene expression analysis, manuscript preparation. NK, SP, and BV experiments investigating the effect of metformin and GW501516 on FFA uptake. MiK gene expression analysis of all samples. ME design and performance of FFA uptake and oxidation experiments, data interpretation. JR confocal microscopy imaging, co-localization analysis, metformin effect on FFA uptake. FD, MaK, supervision and design of Oxygraph measurements, data interpretation. MT cell culture and passaging, lipid content analysis under all experimental conditions. JT and JP design, supervision and coordination of the project, data analysis and manuscript preparation. KB performed western blotting analyses in all experiments. All authors have participated in the manuscript preparation and approved its final form.

### Conflict of interest statement

The authors declare that the research was conducted in the absence of any commercial or financial relationships that could be construed as a potential conflict of interest.

## Abbreviations

ADP: Adenosine diphosphate
AKT: Protein kinase B
BCA: Bicinchoninic acid assay
BSA: Bovine serum albumin
CD36: Cluster of differentiation protein 36
CPAP: Continuous positive airway pressure
CPT-1: Carnitine-palmitoyl transferase-1
DMSO: Dimethylsulfoxide
FBS: Fetal bovine serum
FFA: Free fatty acids
GLUT1: Glucose transporter 1
IRβ: Insulin receptor-β
IRS-1: Insulin receptor substrate-1
OSA: Obstructive sleep apnea
PBS: Phosphate-buffered saline
PGC1α: Peroxisome proliferator-activated receptor gamma coactivator 1-alpha
PMP70: Peroxisome-specific protein 70
PPAR: Peroxisome proliferator-activated receptors
SLC27A4: Solute carrier family 27 (fatty acid transporter), Member 4
T-PER: Tissue-protein extraction reagent
T2DM: Type 2 diabetes mellitus
UCP2: Uncoupling protein 2.
