# Supplementary figures and images for: The Effect of Hypoxia and Metformin on Fatty Acid Uptake, Storage, and Oxidation in L6 Differentiated Myotubes
Source: Front Endocrinol (Lausanne). 2018 Oct 17;9:616. doi: 10.3389/fendo.2018.00616 (PMC6199370; doi:10.3389/fendo.2018.00616)

FATP4 7day

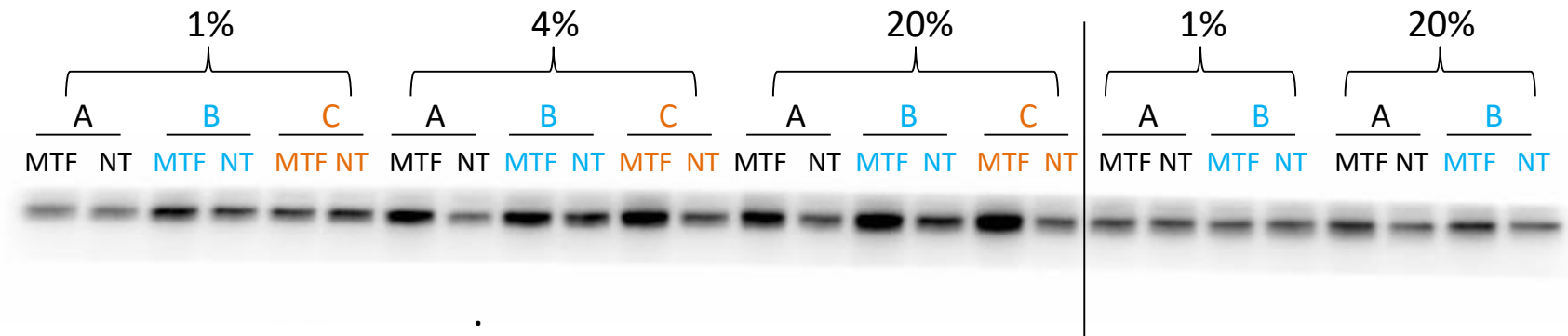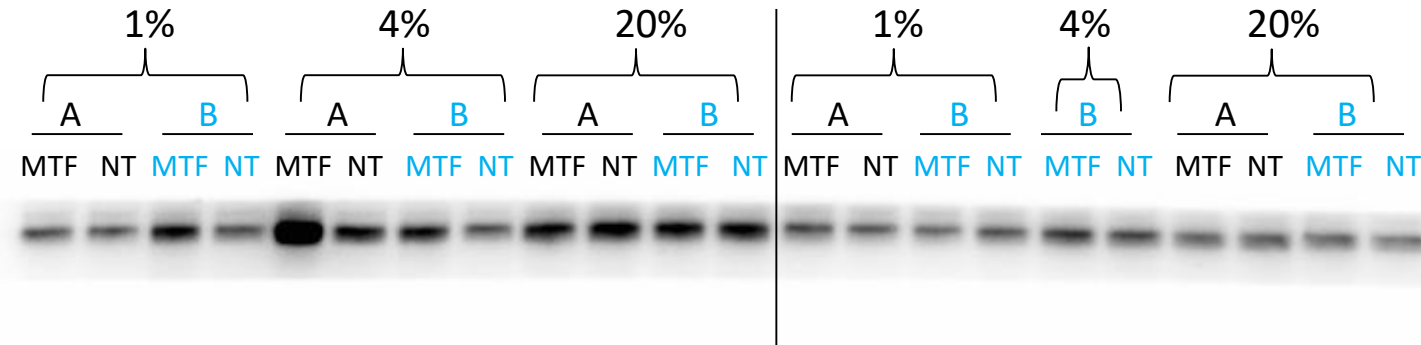

GAPDH for FATP4 7days

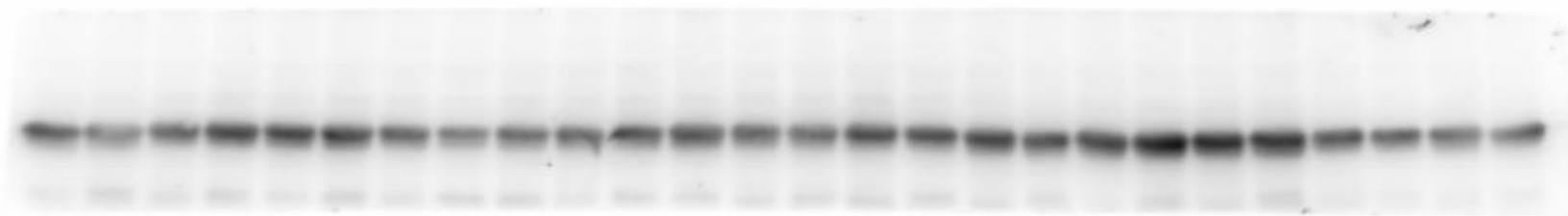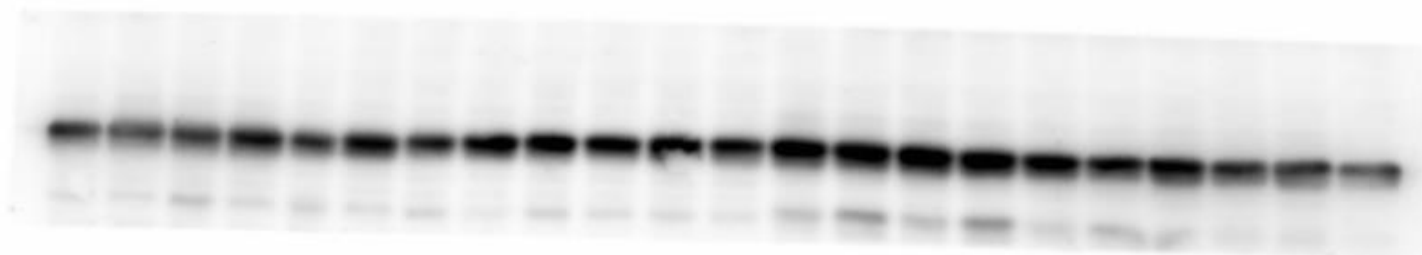

CD36 7days

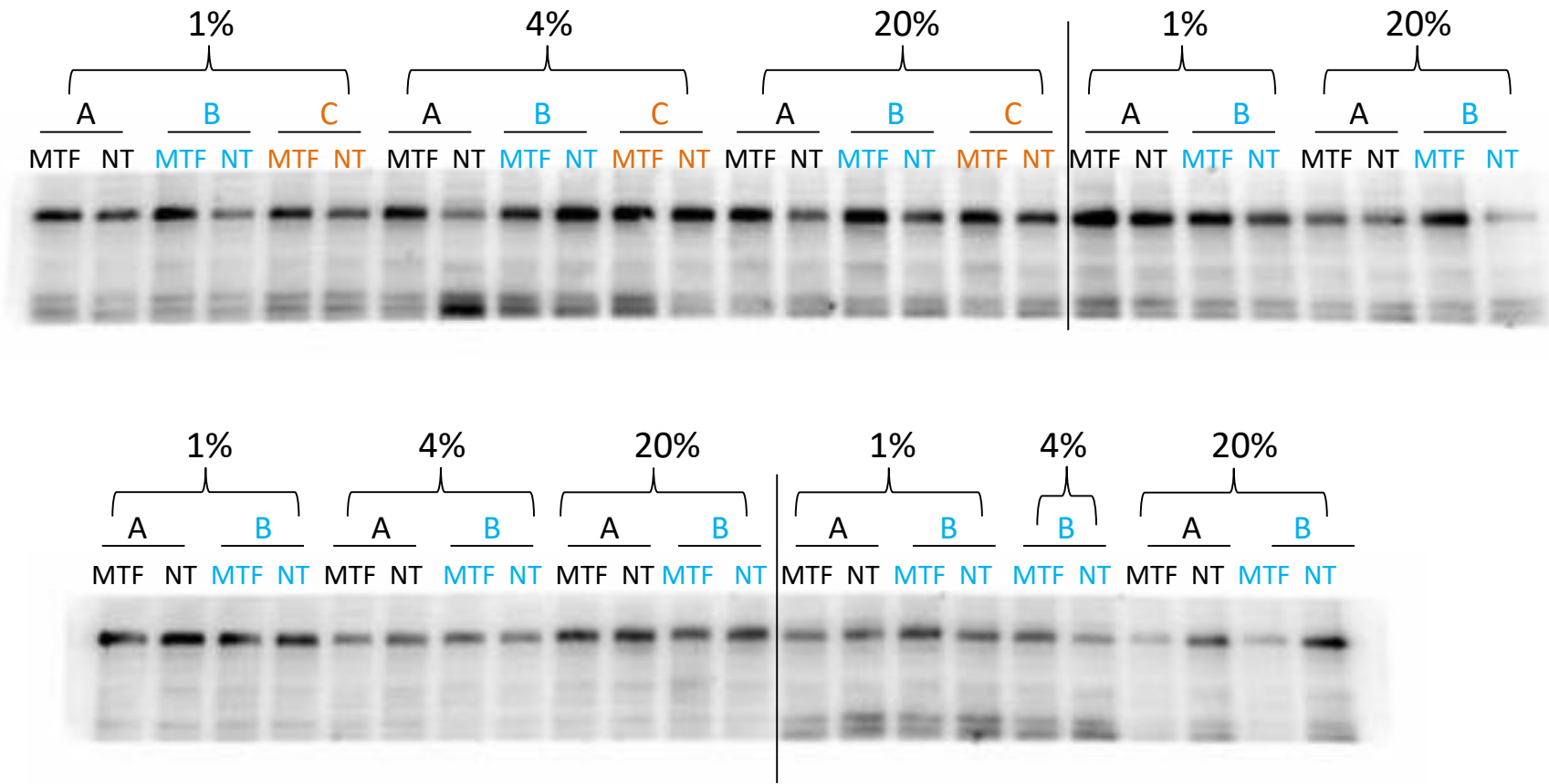

GAPDH for CD36 7 days

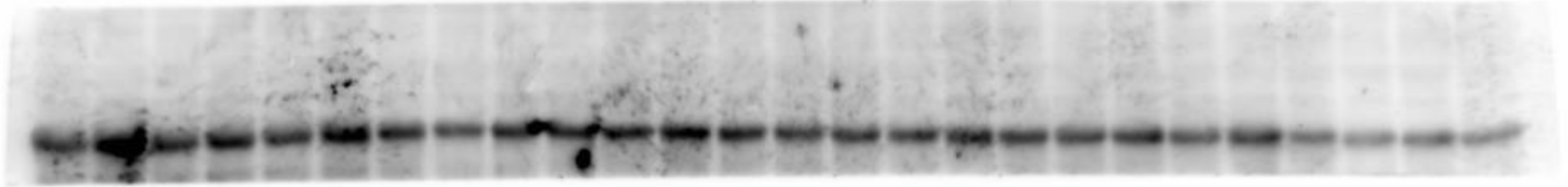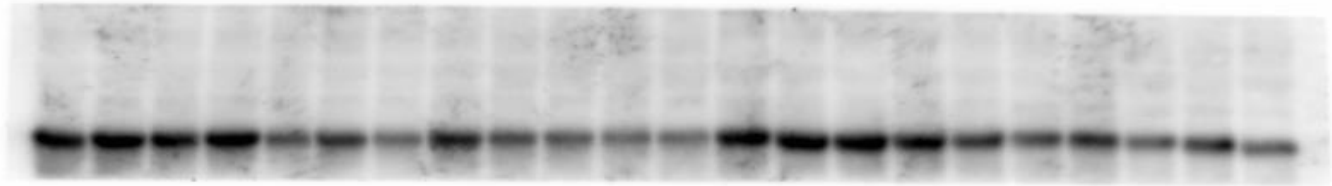

Supplement: Supplementary file 2 [file Data_Sheet_1.pdf]
